# Enhancing the Antibiotic Production by Thermophilic Bacteria Isolated from Hot Spring Waters via Ethyl Methanesulfonate Mutagenesis

**DOI:** 10.3390/antibiotics12071095

**Published:** 2023-06-23

**Authors:** Yasmin G. Kortam, Wafaa M. Abd El-Rahim, Abd El-Nassar A. Khattab, Nazih Y. Rebouh, Regina R. Gurina, Olfat S. Barakat, Mohamed Zakaria, Hassan Moawad

**Affiliations:** 1 Department of Agricultural Microbiology, National Research Centre, Giza 12622, Egypt; yg.gamal@nrc.sci.eg (Y.G.K.);; 2 Department of Genetics and Cytology, National Research Centre, Cairo 12622, Egypt; 3 Department of Environmental Management, Institute of Environmental Engineering, RUDN University, 6 Miklukho-Maklaya Street, 117198 Moscow, Russia; rebukh_nya@pfur.ru; 4 Technosphere Security Department, RUDN University, 6 Miklukho-Maklaya Street, 117198 Moscow, Russia; 5 Department of Agricultural Microbiology, Faculty of Agriculture, Cairo University, Cairo 12613, Egypt

**Keywords:** antibiotic-resistant bacteria, antimicrobial, harsh environment, ethyl methanesulfonate

## Abstract

Antibiotic-resistant bacteria represent a serious public health threat. For that reason, the development of new and effective antibiotics to control pathogens has become necessary. The current study aims to search for new microorganisms expressing antibiotic production capacity. Fifteen sites covering a wide range of harsh environmental conditions in Egypt were investigated. Two hundred and eighty bacterial isolates were obtained and then tested against pathogenic bacteria using the agar disk diffusion technique. Fifty-two (18.6% of the total) of the isolates exhibited antagonistic properties, which affected one or more of the tested pathogens. The isolate 113 was identified as *Bacillus licheniformis* and isolate 10 was identified as *Brevibacillus borstelensis* using the 16S rRNA technique. The *B. licheniformis* strain was stronger in antibiotic production against *S. typhi, M. luteus*, and *P. ariginosa,* whereas the strain *Br. borstelensis* was more efficient against *B. cereus, E. coli,* and *Klebs.* sp. The sensitivity of the strains to commercial antibiotics showed that *B. licheniformis* was highly sensitive to seven commercial antibiotics, whereas *Br. borstelensis* was sensitive to nine antibiotics. The two strains were subjected to ethyl methanesulfonate (EMS) mutagenesis to obtain mutants with a higher antibiotic production. The total bacterial count was measured after treatment with EMS mutagen and showed a significant gradual increase in the antimicrobial activity, which was achieved via shaking in the presence of EMS for 60 min. High antimicrobial activities were noted with 17 and 14 mutants from the *B. licheniformis* and *Br. borstelensis* strains, respectively. The mutant *B. licheniformis* (M15/Amo) was more active than the parent strain against *S. aureus* (212.5%), while the mutant *Br. borstelensis* (B7/Neo) was more effective against *S. typhi* (83.3%). The present study demonstrates the possibility of obtaining potent antibiotic-producing bacteria in hot spring waters and further improving the indigenous bacterial capacity to produce antibiotics by using EMS mutagenesis.

## 1. Introduction

Antibiotics are small bioactive molecules that are naturally produced by microorganisms like bacteria and fungi during their secondary metabolism [1,2]. In 1941, the term “antibiotics” was first used by Selman Waksman to describe microscopic substances produced by microorganisms that prevent the growth of other germs [3]. The development of penicillin, which is made by a fungus, as well as the development of streptomycin, chloramphenicol, and tetracycline, which are made by soil bacteria, initiated the antibiotic era between 1945 and 1955 [4]. These original antibiotics are now mainly ineffective due to the evolution of antibiotic resistance by significant human diseases, and if replacements are not discovered, the golden age of antibiotics will soon come to an end [5,6].

The increasing prevalence of antibiotic resistance reduced or abolished the in vivo efficacy of antibiotics, and the spread of antibiotic-resistant microorganisms is now threatening the treatment of infectious diseases [7,8]; therefore, the need for research on antimicrobial agents to overcome this serious threat to human, animal, and plant health is becoming a priority to control the spread of antibiotic-resistant pathogens [9] that cause life-threatening diseases [10].

It is known that terrestrial and aquatic environments represent the natural reservoir of a wide range of microorganisms that interact with each other [11,12]. Microbial metabolites produced in this interaction are rich sources of new potential bioactive compound [13,14].

The antimicrobial activity of certain bacteria represents an important tool for defending the microbial persistence of many microorganisms in soil and water [15,16,17].

A good method for obtaining novel bioactive compounds is to harbor antibiotic activity [18,19]. Only about 100 antibiotics have been used commercially out of the 5000 antibiotics produced by the cultures of Gram-positive bacteria, Gram-negative bacteria, and filamentous fungus [20]. To combat the resistance to the currently available antibiotics, researchers must look for less harmful, more effective antibiotics from non-infectious organisms [21]. The bacteria that have evolved to withstand situations with minimal water availability, high salinity, extreme temperatures, and pH could be sources of novel bioactive chemicals [22,23]. The aquatic and terrestrial ecosystems in Egypt are rich in native microbes, many of which are tolerant to various stress conditions [23,24,25].

Numerous studies were conducted to optimize the microbial culture conditions to increase the effectiveness of antibiotics [26] using genetic improvement protocols [27]. Chemical mutagenesis via ethyl methanesulfonate (EMS) was used to obtain stable mutants with enhanced antimicrobial production [28].

The aim of this work is to search for potent antibiotic-producing bacteria inhabiting extreme environments and to improve the antibiotic production by promising strains that are capable of producing antibiotics against certain pathogenic bacteria.

## 2. Results

### 2.1. Hot Spring Water and Saline-Affected Soils Used for Microbial Isolation

The temperature of seven hot springs recorded from Sinai, Wadi El-Natrun, and Dakhala and Kharga Oasis ranged between 50 and 90 °C, with samples from the Ras Sedr sulfur hot springs being the highest (90 °C), and Hammam Pharaon being the lowest (50 °C). The electric conductivity of the water sample was measured in the range of 7.05–9.66 ds/m in samples obtained from Sinai and Wadi El-Natrun hot springs. However, in the samples collected from the Oasis sites, the EC was 8.16 in the Dakhala Oasis sample and 9.66 ds/m in the Kharga Oasis hot spring sample. These two samples showed the lower value of dissolved cations and anions compared with all other spring water. The dissolved Na+ was high in the samples from Wadi El-Natrun. The data in Table 1 show a wide range of the chemical properties of hot spring waters collected from the geographical zones in Egypt.

Regarding the analysis of soils affected by hot spring water and salinity conditions, the temperatures of the soils around hot springs were as high as the hot spring water (50–90 °C). The electric conductivity and sodium content of the Wadi El-Natrun lake soil and soil collected from the rhizosphere of plants growing in the Suez Gulf coastal area in Sinai, the hot spring water, and the salt-affected soils were selected to cover a wide range of microbial conditions to obtain the diversity of microbes needed for further testing of antibiotic production under these harsh conditions. 

### 2.2. Isolation of Thermophilic and Halophilic Microbes

Fifteen sites covering a wide range of extreme conditions were explored for the presence of antibiotic-producing microbes. These sites included 11 hot spring sites at Sinai, Wadi EL-Natrun, Suez Gulf, Hammam Pharaon, Ras Sedr, and Dakhla and Kharga Oasis at New Valley. Four additional sites were included for the isolation of the antibiotic-producing microbes from salt-affected soils at Sinai, Wadi El-Natrun, and the Suez Gulf coastal zone. The total isolates from all 15 of the above mentioned samples totaled 280 bacterial isolates, as seen in Table 2. Based on the zone of inhibition observed on agar plates, antibiotic-producing microbes were screened. Out of 280 bacterial isolates, only 52 bacterial isolates were antibiotic-producing bacteria. The phenotypic differentiation among the fifty-two antimicrobial-producing bacterial isolates based on colony and cell morphology as well as Gram staining showed that all of the isolates were Gram positive, except for isolates No. 8, 118, 131, 135, and 184, which were Gram negative. The contents of the 52 antibiotic-producing bacterial isolates were found to be bacteria of the bacilli except for those found in isolates No. 19, 122, 130, 191, and 229, which were streptococci based on their morphological appearance, as observed under the microscope. 

### 2.3. Screening Isolates for Antibiotic Production Using Standard Agar Disc Diffusion (SADD) Method

**Figure 1:**
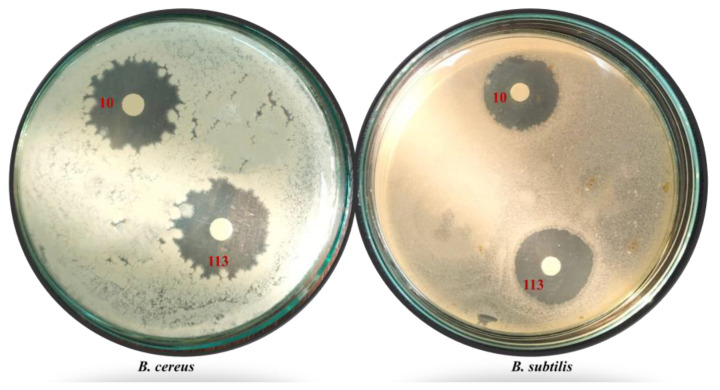
Inhibition zones generated by bacterial isolates No. 10 and 113 against *S. typhi* and *B. sereus* pathogenic test microorganisms (four replicates each treatment).

The testing of these isolates against eight pathogenic test bacteria showed that only 52 isolates (18.6%) were capable of showing antagonistic activity against one or more of the pathogenic test organisms. The antibiotic-producing bacteria collected from hot water samples amounted 38 isolates (13.6%) of the total bacterial isolates, whereas the antibiotic-producing isolates from soils did not exceed 5% of the total. Some hot spring water samples were marked by a higher percentage of isolates that produce antibiotics. In the samples from the Dakhla Oasis, Hammam Pharaon hot spring, Ras Sedr hot springs, and Kharga Oasis hot spring, the percentage of antibiotic-producing bacteria amounted 46.1, 33.3, 29.4, and 26.3% of the isolates from these tested samples, respectively. Regarding the presence of antibiotic-producing bacteria in soils affected by hot spring water and salinity conditions, the isolates producing at the highest percentages of the total isolates from the same site were from the soil affected by the Hammam Pharaon hot spring water at 28.5% followed by 15.7% among all isolates from the rhizosphere sample affected by the Suez Gulf saline water (Table 2, Figure 1).

The antibiotic production profiles (APP) of the fifty-two isolates collected from the hot spring water and soils affected by hot spring waters and saline conditions are presented in Table 3. According to the research, the isolates from the hot spring waters were often more capable of producing antibiotics than those isolated from saline soils. All isolates were divided into 35 antibiotic production profiles, including 24 from hot water sources and 11 from soils affected by hot waters and salinity. The broad-spectrum range of several isolates from hot waters was higher than those from soils (Table 3). From the first nine sites of hot water sampling, 13 isolates antagonized six pathogenic tests. However, only one soil sample affected by the hot spring water yielded two isolates that were capable of antagonizing seven pathogenic tests. Isolates 10 and 113 isolated from the Kharga Oasis and Hammam Pharaon hot springs were the broadest spectrum, as all the test pathogens were killed by the antibiotics produced by these isolates, with a wide diversity of isolates in relation to antibiotic production. The least active isolate was from the soil affected by the Hammam Pharaon hot water, where only the isolate 156 was capable of antagonizing *B. subtilis* (Table 3).

### 2.4. Molecular Identification of the Potent Antibiotic-Producing Bacterial Isolates 10 and 113

Nearly full-length 16S rRNA gene products (1500 bp) were amplified and sequenced from the potent antibiotic-producing bacterial isolates 10 and 113. The sequences of the 16S rRNA gene were blasted to the EZBioCloud database. The sequences of the 16S rRNA gene from isolates 113 shared 97.83% similarity with *Bacillus licheniformis* strain AT59. The 16S rRNA sequence of isolate 10 displayed a high similarity of 97.41% with *Brevibacillus borstelensis* MA-49 (KX426649.1).

**Figure 2:**
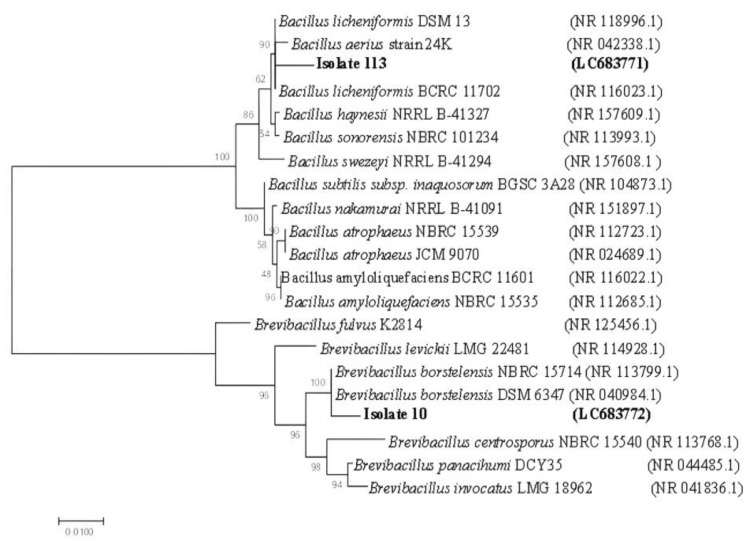
Neighbor-joining (NJ) tree based on 16S rRNA sequences, showing the relationships between hot spring water antibiotic-producing strains and recognized species of *B. licheniformis* and *Br. borstelensis*. Bootstrap values are indicated for each node (1000 replicates). Sequences retrieved in this study are shaded.

Based on the ML phylogenetic tree utilizing 16S rRNA sequences, the bacterial isolates were closely affiliated to two genera, *Bacillus* and *Brevibacillus*, and were grouped in two distinct clusters (Figure 2). Due to the low phylogenetic power at the species level, the newly isolated bacteria in this study were assigned only to the genus level, isolate 113 was identified as *Bacillus* sp. and tightly grouped with type strains of *Bacillus licheniformis, Bacillus aerius, Bacillus haynesii, Bacillus sonorensis*, and *Bacillus swezeyi*, supported by a 100% BT value. Isolate No. 10 was assigned to *Brevibacillus* sp. and grouped with species of *Brevibacillus fulvus, Brevibacillus levickii*, *Brevibacillus borstelensis*, *Brevibacillus centrosporus*, *Brevibacillus panacihumi*, and *Brevibacillus invocatus* (100% BT, 99.6% ANI).

### 2.5. Antimicrobial Inhibitory Effect of Two Potent Bacterial Supernatants against Test Pathogenic Bacteria

**Figure 3:**
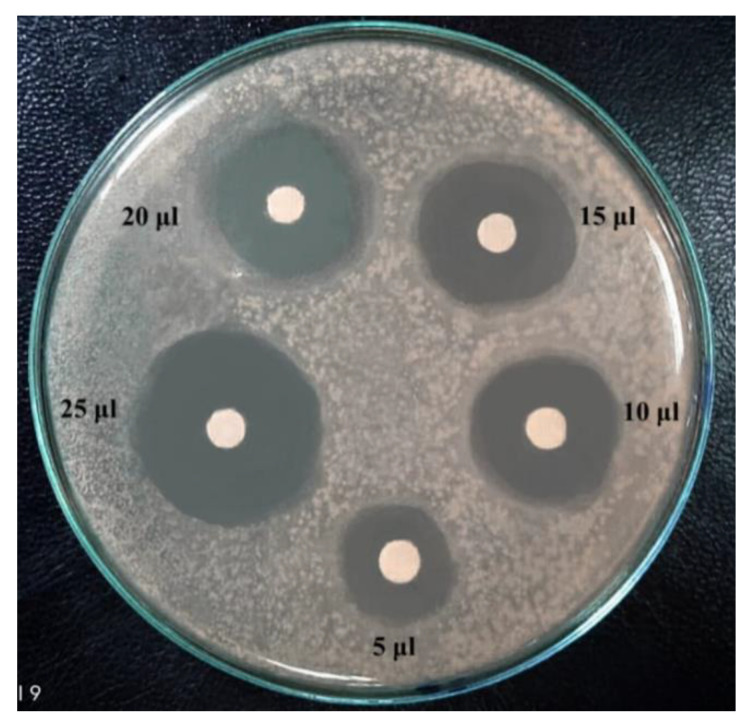
Antimicrobial inhibitory effect of *B. licheniformis* supernatant against *B. cereus*.

The degree of antagonism by the two selected strains, *B. licheniformis* 113 and *Br. borstelensis* 10, isolated from hot spring waters, was further assessed using the antimicrobial inhibitory effect of cultural supernatant against eight pathogenic test bacteria. A total of 25 μL of the cultural supernatant was loaded on filter discs and placed on the growth of the test bacteria Figure 3. The results in Table 4 show that the supernatant of *B. licheniformis* 113 was stronger in the antagonism against *E. coli, K. pneumonia, B. cereus, B. subtilis*, and *P. ariginosa* compared with the supernatant from *Br. borstelensis* 10. The latter was, on the contrary, more potent against *P. aeruginosa, E. coli, B. cereus*, and *M. luteus*. Despite the difference in the antimicrobial inhibitory effect of both strains, the strains seem to have a strong specific inhibitory effect against the tested pathogenic bacteria. 

### 2.6. Mutagenesis Using Ethyl Methanesulfonate (EMS)

Before mutation induction, a good selective method to isolate mutants with heterogeneous genotypes must be found. Here, we used the response to antibiotics as a strong selective method by measuring the resistance and sensitivity of many antibiotic discs. Antibiotic discs were used, which were strong in killing the bacterial strains under study, and gave the largest area of inhibition to serve as detectors for colonies that can grow in large areas of inhibition around such type of antibiotics after the mutation induction via chemical mutagen. The resistance of *B. licheniformis* 113 and *Br. borstelensis* 10 to 13 preloaded commercial antibacterial discs were assessed.

The results show a high sensitivity of *B. licheniformis* 113, measured by the diameter of inhibition zones to rifampicin-SV (26 mm), followed by chloramphenicol (18 mm), polymyxin B (16 mm), norfloxacin (12 mm), gentamycin (11 mm), tetracycline (8 mm), and amoxycillin (8 mm). The strain was resistant to six antibiotics, namely, vancomycin, oxytetracycline, neomycin, kanamycin, streptomycin, and erythromycin (Table 4). In contrast, *Br. borstelensis* 10 was resistant to vancomycin, rifamycin-SV, amoxycillin, and streptomycin. This strain was sensitive to eight antibiotics, especially chloramphenicol (24 mm), followed by norfloxacin (16 mm) and erythromycin (15 mm) (Table 5).

### 2.7. Genetic Improvement of Antimicrobial-Producing Strains via Ethyl Methanesulfonate (EMS) Mutagenesis

The EMS technique was used to perform chemical mutagenesis on *B. licheniformis* 113 and *Br. borstelensis* 10 strains to increase antibiotic production. The mutant antibiotic-producing bacterial strain counts were measured at different shaking periods. The results show that the total number of bacteria gradually decreased with continuous shaking for 60 min. This may be due to the direct toxic effect of EMS on bacterial cells (Table 5). The antibiotic production by living mutant bacterial strains against test pathogenic bacteria at different shaking periods was studied. Table 6 shows a significant gradual increase in antimicrobial activity with continuous shaking for 60 min. This indicates the positive effect of the mutagenic process on the antimicrobial production by the bacterial strains used.

In general, 17 and 14 antibiotic-resistant mutants were selected from the strains *B. licheniformis* 113 and *Br. borstelensis* 10, respectively, and the evaluations of their antibacterial activity are shown in Table 7 and Table 8. The antimicrobial effects of mutagenic *B. licheniformis* 113 against pathogenic test bacteria are shown in Table 7. All the mutants (M 1 to M 17) demonstrated higher activity against all the test bacteria when compared to those that the parental strains did. Mutant M 15 recorded as the highest antimicrobial activity against all pathogenic bacteria, followed by M 8, compared to the parental strains as follows:

$$\left(\mathrm{Efficiency}=\frac{\mathrm{Mutant}\;\mathrm{activity}-\mathrm{Parant}\;\mathrm{activity}}{\mathrm{Mutant}\;\mathrm{activity}}\times 100\right)$$

The improvement range for M 15 was from 59.1 (*E. coli*) to 212.5% (*S. aureus*), while it was from 50 (*B. cereus* and *S. typhi*) to 175.1% (*M. luteus*) for the mutant M8. In contrast, the mutants B7 and B11 represented the highest antimicrobial activities against most pathogenic bacteria, respectively, compared to those of the parental strains. The mutant B7 represented the highest antimicrobial activities against most pathogenic test bacteria ranging from 21.05 (*K. pneumoniae*) to 83.3% (*S. typhi*), while the mutant B11 showed an improvement range from 40.2 (*E. coli*) to 100.1% (*St. aureus*). According to the aforementioned EMS mutant’s data, all the obtained mutants exhibited an improvement in their anti-microbial activity against all pathogenic testers, and the improvement percentage in the case of *B. licheniformis* 113 varied from 312.5 (M15/AMX against to *S. aureus*) to 104.55 (M7/OXY against to *B. subtilis*), whereas in the case of strain *B. borstelensis* 10, the improvement rate ranged from 200 (B11/PM-B against to *S. aureus*) to 105.55 (B3/GEN against to *S. typhi*) (Table 8). 

## 3. Discussion

The need to develop new antimicrobial substances is growing, since many traditional commercial antibiotics have lost their efficiency due to the multidrug resistance of pathogens to widely used antimicrobials. This study is one of the trails used to explore potential new chemical antibiotics, particularly, marginal studies environments, such as places exposed to high temperatures and salinity. This approach was also studied by other authors [29,30,31,32,33].

Seven hot spring water samples and five soil samples, two of which were affected by salinity and three affected by hot spring waters, were used for the isolation of antibiotic-producing microorganisms. Among the 280 isolates obtained from the 15 sites, 52 isolates possessed the capacity to produce antimicrobials against test pathogens used in this study. The ratio of antibiotic-producing microorganisms to non-antibiotic-producing microorganisms is in line with the findings of other authors [34,35]. Regarding the presence of antibiotic-producing bacteria in soils affected by hot spring waters and salinity conditions, the isolates producing at the highest percentages of the total isolates from the same site were from the soil affected by the Hammam Pharaon hot spring water at 28.5% followed by 15.7% among all isolates from the rhizosphere sample affected by the Suez Gulf saline water. Antimicrobial production via thermophilic and halophilic bacteria isolated from extreme conditions was stated in several reports [36,37].

The broad-spectrum range of several isolates from the hot spring waters was higher than those from the soil samples. From the first nine sites of hot water sampling, 13 isolates antagonized six pathogenic tests. However, only one soil sample affected by the hot spring water gave two isolates that were capable of antagonizing seven pathogenic test organisms. Isolates 10 and 113 isolated from the Kharga Oasis and Hammam Pharaon hot springs had the broadest antibiotic spectrum among all of the isolates. The test pathogens used in this study were killed by the antibiotics produced by these isolates. The least active isolate was from the soil affected by the Hammam Pharaon hot spring water, where only the isolate 156 was capable of antagonizing *B. subtilis*. The differences in the antibiotic production by microbes isolated from similar sites were previously stated in [38,39].

The two potent isolates in antibiotic production, isolate No. 113 from the Kharga Oasis water sample and isolate No. 10 from the Hamam Pharaon water springs, were identified using the 16S rRNA technique. The sequences of the 16S rRNA gene from isolate 113 shared 97.83% similarity with the *Bacillus licheniformis* strain AT59, whereas the 16S rRNA sequence of isolate 10 displayed a high similarity of 97.41% with *Brevibacillus borstelensis* MA-49 (KX426649.1).

The supernatant of strain *B. licheniformis* 113 was stronger in the antagonism against *S. typhi, S. aureus, M. luteus*, and *P. ariginosa* compared with the supernatant *from Br. borstelensis* 10. The latter was, on the contrary, more potent against *B. cereus, E. coli*, and *K. pneumonia*. Despite the difference in the minimal inhibitory effect of MIE on both strains, the strains seem to have a strong specific inhibitory effect against the tested pathogenic bacteria. The antimicrobial inhibitory effects of *B. licheniformis* and *Br. borstelensis* were also studied previously [40,41].

The sensitivity of the *B. licheniformis* 113 obtained in this study showed the diameter of the inhibition zones to rifampicin-SV (26 mm), followed by chloramphenicol (18 mm), polymyxin B (16 mm), norfloxacin (12 mm), gentamycin (11 mm), tetracycline (8 mm), and amoxycillin (8 mm). This strain was resistant against six antibiotics, namely, vancomycin, oxytetracycline, neomycin, kanamycin, streptomycin, and erythromycin. The strain *Br. borstelensis* 10 was resistant against vancomycin, rifamycin-SV, amoxycillin, and streptomycin. This strain was sensitive to eight antibiotics, especially chloramphenicol (24 mm), followed by norfloxacin (16 mm) and erythromycin (15 mm). Therefore, the antibiotics were used as markers to select the most resistant mutants [42,43,44].

The genetic improvement in the antibiotic production by two potential strains using EMS mutagenesis was studied. In nature, mutations occur at extremely low rates. Due to the exposure to various mutagenic agents, mutations can occur in changes in the nucleotide sequences of DNA (e.g., point mutations, double mutations, deletions, and insertions). Induced mutagenesis is a common technique for creating new mutant strains with better characteristics. The most common mutagen used to enhance bacterial strains is EMS, which causes a variety of point mutations in the bacterial DNA. Exceptional mutants are formed at increasing quantities as a result of these point modifications, in conjunction with regular adjustments to enhance the antibiotic production [45,46].

The total number of bacteria after treatment gradually decreased with continuous shaking for 60 min. This may be due to the direct toxic effect of EMS on bacterial cells. The antibiotic production by living mutant bacterial strains against test pathogenic bacteria at different shaking periods was studied. A significant gradual increase in the antimicrobial activity with continuous shaking for 60 min was recorded. This indicates the positive effect of the mutagenic process on the antimicrobial production by the bacterial strains used. Antimicrobial stimulation via EMS mutagenesis was stated in previous studies [46,47,48].

McAuley et al. [49] discovered that the possessed resistance genes for the antimicrobial molecules they produce are often related to and coregulated with antibiotic production genes. The overproduction of the secondary metabolite actinorhodin via strR mutations of *S. coelicolor* was reported in [50]; it was also reported by El-Bondkly and Khattab [51], who detected that clavulanic acid and cephamycin improvement in *Streptomyces clavuligerus* happens by promoting combination resistance mutations and protoplast fusion. The antibacterial agents (bacteriocins) of probiotic bacteria were enhanced via UV, NTG, and EMS, and improvements of 290%, 266%, and 345.7% were achieved. El-Sherbini and Khattab [28] found that the lincomycin produced by *Streptomyces lincolnensis* increased by 1.5-fold via the induction of novel mutants using ultra-violet (UV) irradiation and ethyl methanesulfonate (EMS). Finally, Qattan and Khatab [52] demonstrated that genetically enhanced mutants of *Micromonospora echinospora* increased gentamicin production, and mutants of *Streptomyces albogriseolus* improved neomycin production.

The results clearly show that exploring the antibiotic production by native microbes inhabiting the less studied harsh environments is a promising approach for the isolation of potential microbial resources for antibiotic production. The additional genetic improvement in potential strains has proven to be among the techniques for a better production of antimicrobials. The continuous efforts in this direction will pave the way for combating the multidrug resistance phenomenon threatening the life of patients suffering from serious diseases.

## 4. Material and Methods

### 4.1. Collection of Samples from Extreme Conditions

**Figure 4:**
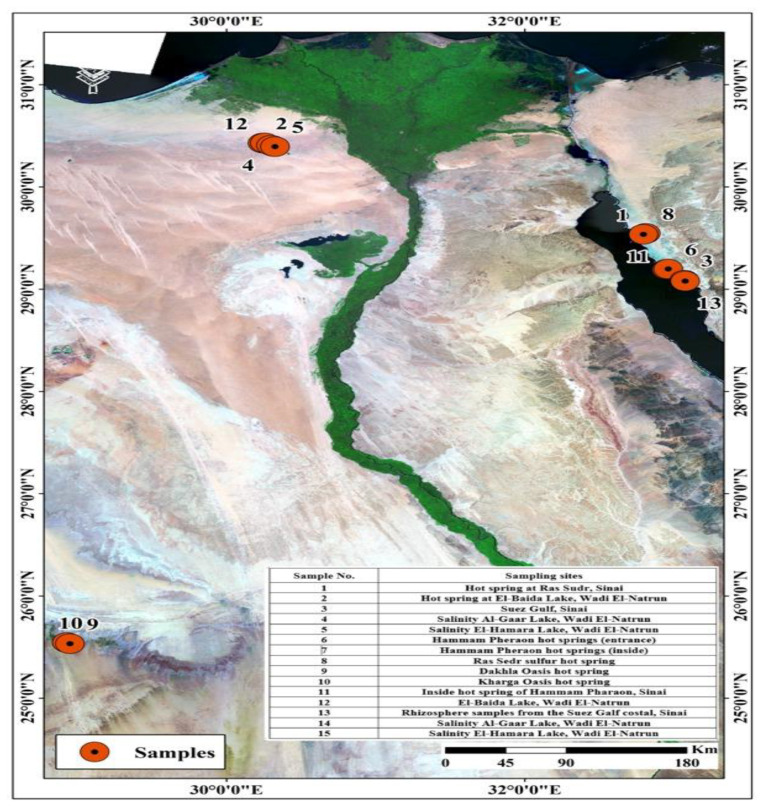
Water sample locations.

Figure 4 shows water samples from hot springs and soils close by that were collected from several locations in Egypt including Hammam Pharaon, the Closed Sulfur Lake at Ras Sedr at Sinai, and the Closed Dakhla as well as Kharga lakes. Samples affected by high salinity were obtained from different lakes, such as Red Lake, Gaar Lake, and Al-Beuda Lake at Wadi-Natrun, Egypt. Samples of hot springs were collected in double-walled containers to maintain the temperature until analysis. Samples of salt lakes were obtained in sterile plastic containers and kept in an ice box and then in a refrigerator at 4 °C until analysis. Using a random sampling technique, soil samples were taken from the study area at a depth of 5–10 cm and kept in sterile polyethylene bags at 4 °C until analysis. All samples were examined according to APHA (American Public Health Association) guidelines [53].

### 4.2. Isolation of Thermophilic and Halophilic Microbes

Using the serial dilution agar plate technique, microbes were recovered from soil and water samples. Nine milliliters of sterile distilled water was combined with one milliliter of each water sample or one gram of each soil sample, and the mixtures were serially diluted up to a 10^−6^ fold dilution. Using the same water source, an aliquot of 0.1 mL of each dilution was used to inoculate the nutrient agar (NA) medium for isolating bacteria, the starch casein (SC) medium was used for isolating actinomycetes, and the potato dextrose agar (PDA) medium was used for isolating fungi. The sterilization process was placed at 121 °C for 20 min. The plates were inoculated using the spread plate method and incubated at 60 °C for 2 days for the thermophilic bacteria, 5 days for thermophilic fungi, and 7 days for thermophilic actinomycetes. Isolation of halophilic bacteria was performed via incubation at 30 °C for 2 days for the halophilic bacteria, 5 days for halophilic fungi, and 7 days for halophilic actinomycetes. From each plate, a single colony was selected and purified via streaking [54,55].

### 4.3. Screening Isolates for Antibiotic Production Using Standard Agar Disc Diffusion (SADD) Method

The microbial isolates were assessed for antibiotic production using the SADD method against 8 pathogenic bacteria. The tested bacterial isolates were the following: *Klebsiella pneumonia* ATCC7000603, *Escherichia coli* ATCC25922, *Bacillus subtilis* ATCC6633, *Bacillus cereus* ATCC14579, *Staphylococcus aureus* ATCC6538, *Micrococcus luteus* ATCC10240, *Pseudomonas ariginosa* ATCC9027, and *Salmonella typhi* ATCC14028. The thermophilic isolates were grown at 50 °C for 48 h on a shaker (120 rpm) in 100 mL of nutrient broth media prepared by using the same original source of water. The halophilic microbes were grown at 30 °C. The test strains were grown in 100 mL of nutrient broth media and incubated at 37 °C for 24 h. The optical density of broth cultures was determined using the UV–VIS spectrophotometer (Bio Chrome Libra S70) at λ_600_ nm to standardize microbial growth above 0.6. Fungal filtrates were separated via filtration using Whatman, No. 0.1, whereas bacterial culture supernatant was separated via centrifugation (6000 rpm, 4 °C, 15 min). To ensure that the bacteria were evenly distributed across the agar medium, 1 ml of the bacterial test strain inoculum suspension was spread over the media using a sterile glass rod. Twenty microliters of microbial isolates filtrates/supernatants was loaded on paper discs. Paper diffuse discs were made from the media and then arranged on the plate. The plate was then transferred to an incubator at 37 °C for 24 h after being incubated at 5 °C for an hour to promote excellent diffusion. By employing a zone reader to measure the size of the clear inhibitory zone surrounding the disc, the antibacterial activity was captured (mm) [56,57].

### 4.4. DNA Isolation and Molecular Identification of Bacterial Isolates

GeneJet^TM^ Genomic DNA Purification Kit was used to extract the whole genomic DNA from bacterial cells (Thermo Scientific^®^, Waltham, MA, USA). The procedures were carried out in accordance with the manufacturer’s instructions. Bacterial 16S rDNA was amplified using 27F: 5″–AGAGTTTGATCCTGGCTCAG-3″ and 1492R: 5″GGTTACCTTGTTACGACTT-3″ primers [32,33]. The T100 Thermal Cycler was used to conduct the polymerase chain reaction (PCR) (Bio-Rad, Hercules, CA, USA) using the standard reaction mix (25 µL) containing, 1× PCR buffer, 200 mM of each DNTPS, 15 pmol of each primer, 1-unit Taq polymerase enzyme (Promega^®^ Corporation, Madison, WI, USA), 1.5 mM MgCL2, and 50 ng DNA template. The following thermal cycling parameters were used: initial denaturation at 94 °C for 5 min; 30 cycles of 94 °C for 1 min; 55 °C for 1 min; 72 °C for 1 min; and final elongation at 72 °C for 10 min. Using the QIAquick PCR purification kit from Qiagen (Hilden, Germany), 16S rDNA PCR products were purified before being sequenced at Macrogen Inc., Republic of Korea [58,59,60].

### 4.5. Phylogenetic Analysis

Using the software DNA STAR, sequence readings were reassembled together (Lasergene, Madison, WI, USA). The taxonomical identification of bacterial isolates was made to the genus level by blasting partial 16S rDNA gene sequences at EzBioCloud (http:l/eztaxon-e.ezbiocloud.net, accessed on 1 April 2020) databases. With the use of Clustal W version, the acquired sequences were aligned at 1.8 [61]. The MEGA X software’s maximum likelihood (ML) algorithm was used to create the 16S rDNA phylogenetic tree [62] using the Tamura-Nei model (q). With 1000 repeats, bootstrap (BT) support for each node was assessed. The percentage of average nucleotide identity (ANI) between tested isolates and closely related reference strains was calculated using the MEGA X software.

### 4.6. Antimicrobial Inhibitory Effect of Two Potent Bacterial Supernatants Obtained from Hot Spring Waters against Test Pathogenic Bacteria

Agar disc diffusion method was carried out for the determination of the antimicrobial inhibitory effect of the two selected potent bacterial strains obtained from hot spring water. An amount of 25 μL bacterial cultures supernatant were loaded on each paper disc. Test bacterial cultures were inoculated and incubated at 37°C for 24 h. The positive control was chloramphenicol, a broad-spectrum antibiotic against pathogenic bacteria, while the negative control was distilled water. The clear zone diameters around discs were measured [63,64].

### 4.7. Mutagenesis Using Ethyl Methanesulfonate (EMS)

A total of 5 ml of 48 h old culture of *B. licheniformis* 113 and *B. borstelensis* 10 were separately cultivated in nutrient broth and centrifuged (5600 rmp, 4 °C, 3 min). EMS was added to the cell suspension to bring the concentration to 200 mM after the cells were resuspended in 5 mL of sodium phosphate buffer (pH 7.0, 50 mM). For 20, 40, and 60 min, tubes were agitated (100 rpm) at 30 °C. To neutralize the EMS, 500 l of sodium thiosulfate (0.4 M) was then added to the tubes. Centrifugation at (5600 rmp, 10 min) at 4 °C was performed to harvest the cells, which were then washed twice with the same buffer. After being resuspended in phosphate buffer (pH 7.0, 50 mM), the cell pellets were seeded at the proper dilutions (10^−1^ to 10^−6^) on the surface of nutrient agar plates. The two chosen powerful strains and/or their EMS mutants were inoculated onto plates, which were then cultured at 50 °C for 48 h using the standard commercial discs loaded with 13 antibiotics. The antibiotic discs used in this study were loaded with the following antibiotics: vancomycin, gentamycin, oxytetracycline, neomycin, tetracycline, rifamycin-SV, polymyxin, norfloxacin, amoxycillin, kanamycin, streptomycin, erythromycin, and chloramphenicol [65].

The new growth of small colonies in the inhibition zones formed around certain antibiotic discs in plates inoculated with bacterial strains treated with EMS were picked and transferred to nutrient agar plates, as they are considered as mutants resistant to this specific antibiotic.

### 4.8. Assessment of Antimicrobial Activity of the Mutants of B. licheniformis 113 and Br. borstelensis 10

On nutrient agar plates, the isolated colonies of the mutant bacteria were tested against the pathogen microorganisms used before. Each 24 h old (50 µL) pathogenic microorganism was spread and plated after suspension. The discs impregnated with bacterial culture supernatant of the mutants of both bacteria grown for 48 h and centrifuged at 6000 rmp, at 4 °C, for 15 min were placed on the surface of the nutrient agar medium. Each test plate was comprised of four discs. Two mutagen culture supernatant-treated discs were placed at about equal distances from each other on the same plate. Standard antibiotic discs included vancomycin for *B. cereus* and *S. aureus*. Tetracycline was used *for B. subtilis*, *M. luteues*, and *P. ariginosa*. Amoxicillin was used for *E. coli*. Ciprofloxacin was used for *S. typhi*, while penicillin was used for *Klebs*. sp. The negative control was 100% DMSO. After that, the plate was incubated for 24 h at 37 °C. The plates were measured for an inhibitory zone diameter after the incubation. For each test, three replicas were employed [66].

### 4.9. Statistical Analysis

Data were tested for homogeneity of variance using Bartlett’s test, and analysis of variance was used to further evaluate the results (ANOVA). When the ‘F’ test of the ANOVA for treatment was significant at least at the 0.05 probability level, the means were statistically separated on the basis of Duncan’s test (CoHort Software, CoStat version 6.451).

## 5. Conclusions

Multidrug-resistant pathogenic microorganisms are becoming a serious problem, posing a threat to human health; therefore, there is a need to explore various environmental resources to find microorganisms that are capable of producing antibiotics in sufficient amounts to replace the available antibiotics that lost the efficiency due to the multidrug resistance phenomenon. Harsh environmental conditions are known to allow for the special biodiversity of microbes, and many of them may have potential bioactive compounds. This study explores the presence of potent microbial strains in fifteen sites that are exposed to harsh conditions in a trial to find microbes that are capable of producing antibiotics against eight pathogenic test bacteria. A total of 280 bacterial isolates were obtained from hot spring waters and soils affected by saline conditions. The antibiotic production array showed that 52 isolates (19%) produced antibiotics against one or more of the test bacteria. Two isolates identified as *B. licheniformis* 113 and *Br. borstelensis* 10 were potent in the production of antibiotics. Further studies were conducted to enhance the production of antibiotics by these two strains using EMS mutagenesis. The mutants resulting from this mutagenesis step drastically enhanced the antibiotic production against the eight pathogenic test bacteria. This clearly shows the possibilities of obtaining the potent antibiotic-producing bacteria from the harsh environmental sites and the potential of further improving the antibiotic production via strain mutagenesis.

## Figures and Tables

**Table 1:** Physical and chemical analysis of waters collected from hot springs and soils affected by hot spring waters and salinity conditions.

Sample Type	Sample No.	Sampling Sites	Temp. °C	EC dS/m	Dissolved Cations mEq/L				Dissolved Anions mEq/L		
					Ca^2+^	Mg^2+^	Na^+^	K^+^	HCO^3−^	Cl^−^	SO_4_^2−^
Water	1	Hot spring at Ras Sedr, Sinai	90	7.05	13.2	53	30	131	2.16	130	83.34
	2	Hot spring at El-Baida Lake, Wadi El-Natrun	60	8.16	17.58	8	11.5	21	1.8	17	23.7
	3	Suez Gulf, Sinai	25	7.68	63.1	31	138	745.3	1.8	643	274.1
	4	Salinity Al-Gaar Lake, Wadi El-Natrun	28	9.27	177	130	190	1456	110	1620	53.2
	5	Salinity El-Hamara Lake, Wadi El-Natrun	28	8.02	329	55	116.5	2488	39.6	2592	34.1
	6 *	Hammam Pheraon hot springs (entrance)	50	7.07	25.1	62	28	220.3	0.9	250	62.5
	7 *	Hammam Pheraon hot springs (inside)	55	7.69	27.5	84	44	268.1	1.3	285	113.2
	8 *	Ras Sedr sulfur hot spring	90	7.05	15.37	47	16	101	0.7	114	51.4
	9 *	Dakhla Oasis hot spring	51	8.16	4.28	8	11.5	21	1.8	17	23.7
	10 *	Kharga Oasis hot spring	55	9.66	0.71	2	2	3.5	1.3	4	2.32
Soils	11	Inside hot spring of Hammam Pharaon, Sinai	55	7.78	29.2	89	47	287.6	1.9	321	124
	12	El-Baida Lake, Wadi El-Natrun	60	9.66	19.12	2	2	3.5	1.3	4	2.32
	13	Rhizosphere samples from the Suez Gulf coast, Sinai	30	7.83	65.4	38.4	146	873	2.9	759	298
	14	Salinity Al-Gaar Lake, Wadi El-Natrun,	28	9.04	181	136	197	1489	117	1690	61.5
	15	Salinity El-Hamara Lake, Wadi El-Natrun,	28	8.18	358	61	127	2617	43.8	2619	38.5

* Due to the unique properties of the geothermal water samples and, consequently, the prevailing microbiota, the analysis of the samples was made accessible to several researchers exploring the biotechnological potentials of the thermophilic microorganisms. The collected water samples 6, 7, 8, 9, and 10 were analyzed in the laboratory of Egyptian Microbial Culture Collection Network (EMCCN) at the Egyptian National Research Center branch (http:emccn.eg.net, accessed on 1 October 2019).

**Table 2:** Antibiotic-producing bacterial isolates from hot spring waters and saline soils.

Sample Type	Sample Sites	Sample Number	Production of Antibiotics against 8 Pathogenic Test Microorganisms *		17
			Positive	Negative	
Water	Ras Sedr hot spring, Sinai	1	5	12	17
	El-Baida Lake hot spring, Wadi El-Natrun	2	3	10	13
	Suez Gulf, Sinai	3	4	13	17
	Salinity Al-Gaar Lake, Wadi El-Natrun	4	1	17	18
	Salinity El-Hamara Lake, Wadi El-Natrun	5	3	21	24
	Hammam Pheroon hot springs (entrance)	6	5	7	12
	Hammam Pheroon hot springs (inside)	7	4	11	15
	Ras Sedr sulfur hot spring	8	2	16	18
	Dakhla Oasis hot spring	9	6	7	13
	Kharga Oasis hot spring	10	5	14	19
Soils	Hammam Pharaon hot spring, Sinai	11	7	18	25
	El-Baida Lake, Wadi El-Natrun	12	2	17	19
	Rhizosphere samples from the Suez Gulf coast, Sinai	13	3	16	19
	Al-Gaar Lake, Wadi El-Natrun,	14	1	23	24
	El-Hamara Lake, Wadi El-Natrun,	15	1	26	27
Totals	-	-	52	228	280

* Pathogenic test microorganisms are *Klebsiella pneumonia*, *Escherichia coli*, *Bacillus subtilis*, *Bacillus cereus*, *Staphylococcus aureus*, *Micrococcus luteus*, *Pseudomonas aeroginosa*, and *Salmonella typhimurium*.

**Table 3:** Antibiotic production profiles (APP) of thermophilic and halophilic isolates against 8 pathogenic test microorganisms.

Isolate Source	Isolate Number	Sample Sites	Pathogenic Test Bacteria								Spectrum of Antagonism against 8 Pathogens
			*B. cereus*	*B. subtilis*	*E. coli*	*S. typhi*	*Klebs sp.*	*S. aureus*	*M. luteus*	*P. ariuginosa*	
Water	10	Kharga Oasis 1 *	+	+	+	+	+	+	+	+	8
	113	Hammam Pheraon 1	+	+	+	+	+	+	+	+	8
	118, 116	El-Hamara Lake 1	−	+	+	+	+	+	+	+	7
	9, 11	Dakhla Oasis 1	−	+	−	+	+	+	+	+	6
	131, 124	Ras Sedr 1	+	+	+	+	+	+	−	+	7
	111	Hammam Pheraon 1	−	+	+	+	+	−	+	+	6
	109, 108	Suez Gulf 1	−	+	+	+	−	+	+	+	6
	19	Dakhla Oasis 1	+	+	+	+	+	−	+	−	6
	112	Hammam Pheraon 1	+	+	+	+	+	−	+	−	6
	121, 123	Hammam Pheraon 2*	−	+	+	−	+	−	+	+	5
	123, 149	Suez Gulf 1	+	+	+	−	−	−	+	−	4
	114, 126	El-Baida Lake 1	−	−	+	−	+	+	+	+	5
	120	El-Hamara Lake 1	−	−	+	−	+	+	+	+	5
	122	Ras Sedr 1	−	−	+	−	+	+	+	+	5
	22, 20, 13	Dakhla Oasis 1	−	−	+	−	+	+	+	+	5
	119, 124	Hammam Pheroon 1	−	+	−	+	+	−	+	+	5
	115, 125	Ras Sedr 1	−	+	−	+	+	−	+	+	5
	3, 8	Kharga Oasis 1	+	+	+	−	−	−	+	+	5
	25	Ras Sedr sulfur 2	−	−	−	+	+	+	+	+	5
	141, 143	Hammam Pheraon 2	−	−	−	+	+	+	+	+	5
	191	Ras Sedr sulfur 2	−	−	−	+	+	+	+	+	5
	155	Al-Gaar Lake 1	+	−	−	+	−	−	−	−	2
	147	El-Baida Lake 1	+	+	−	−	−	−	−	−	2
	1, 5	Kharga Oasis 1	−	−	+	−	−	−	−	−	1
Soils	136, 138	Hammam Pharaon 3*	−	−	−	+	+	+	+	+	5
	130	Al-Gaar Lake 2	+	+	+	−	−	−	+	−	4
	135, 127	Hammam Pharaon 3	+	+	−	+	+	+	+	+	7
	225	El-Baida Lake 2	+	+	+	−	−	−	+	−	4
	128	Hammam Pharaon 3	−	−	+	−	+	+	+	+	5
	158	Suez Gulf coast 2	−	−	+	−	+	+	+	+	5
	229	El-Baida Lake 2	−	−	+	−	+	+	+	+	5
	182, 23	Hammam Pharaon 3	+	+	−	−	+	+	−	−	4
	157, 154	Suez Gulf coast 2	−	+	+	−	−	−	+	−	3
	184	El-Hamara Lake 2	+	−	−	+	−	−	−	−	2
	156	Hammam Pharaon 3	−	+	−	−	−	−	−	−	1

* 1,2,3 is number of sample sites, + is positive inhibition, − is negative inhibition.

**Table 4:** Antimicrobial inhibitory effect of culture supernatant (25 μL) obtained from *B. licheniformis* 113 and *Br. borstelensis* 10 against 8 test pathogenic bacteria.

Test Bacteria	MIE of Bacterial Culture Supernatant (μL) Containing (AM)	
	*B. licheniformis* 113	*Br. borstelensis* 10
*B. cereus*	15	5
*B. subtilis*	10	10
*E. coli*	15	10
*S. typhimurium*	10	15
*K. pneumonia*	20	10
*S. aureus*	5	10
*M. luteus*	10	15
*P. aeruginosa*	5	25

**Table 5:** Susceptibility of *B. licheniformis* 113 and *Br. borstelensis* 10 to 13 commercial antibiotics.

Bacterial Strains	Diameter of Inhibition Zone (mm)												
	VAN ^+^30	GEN 10	OTC 10	NEO 5	Tet 30	R-SV 30	PM-B 300	NOR 10	AMX 25	KAN 30	STR 10	ERY 15	C 10
*B. licheniformis*, 113	0	11	0	0	8	26	16	12	8	0	0	0	18
*Br. borstelensis*, 10	0	8	12	8	9	0	9	16	0	13	0	15	24

VAN, vancomycin; GEN, gentamycin; OTC, *oxytetracycline;* NEO, neomycin; Tet, tetracycline; R-*SV, rifamycin*-*SV;* PM-B, *polymyxin B;* NOR; norfloxacin; AMX, amoxycillin; KAN, kanamycin; STR, streptomycin; ERY, erythromycin; C, chloramphenicol. ^+^ Concentration (µg/disc). The values are means of diameter of inhibition zones (mm) around discs.

**Table 6:** Total plate count (CFU) of mutant bacterial strains by EMS-mutagenesis at different shaking period.

Period (min)	*B. licheniformis* 113		*Br. borstelensis* 10	
	Count CFU/mL	Survival %	Count CFU/mL	Survival %
0	78 × 10^6^	100	169 × 10^6^	100
20	45 × 10^6^	57.7	62 × 10^6^	36.6
40	38 × 10^6^	48.8	14 × 10^6^	8.3
60	11 × 10^6^	41.2	8 × 10^6^	4.8

**Table 7:** Antagonistic activity of mutant strain *B. licheniformis* 113 against the test bacteria.

Mutant Strains	Inhibition Zone (mm) with Test Pathogenic Bacteria							
	*B. cereus*_a_	*B. subtilis*_b_	*E. coli*_a_	*S. typhi*_c_	*K. pneumonia*_b_	*S. aureus*_d_	*M. luteus*_c_	*P. aeruginosa*_a_
Parent ^h^	22 ^l^_a_ ± 0.05	19 ^m^_c_ ± 0.00	22 ^j^_a_ ± 0.00	10 ^j^_e_ ± 0.05	17 ^l^_d_ ± 0.00	8 ^l^_f_ ± 0.05	8 ^k^_f_ ± 0.00	21 ^l^_b_ ± 0.00
* M1/Van ^ef^	31 ^d^_a_ ± 0.05	24 ^i^_d_ ± 0.00	28 ^e^_b_ ± 0.05	16 ^e^_f_ ± 0.00	23 ^g^_e_ ± 0.00	13 ^g^_g_ ± 0.05	11 ^j^_h_ ± 0.00	26 ^h^_c_ ± 0.05
M2/Van ^fg^	26 ^h^_b_ ± 0.00	26 ^g^_b_ ± 0.00	24 ^h^_d_ ± 0.00	13 ^g^_e_ ± 0.05	28 ^d^_a_ ± 0.00	9 ^k^_f_ ± 0.00	13 ^i^_e_ ± 0.05	25 ^i^_c_ ± 0.00
M3/Van ^g^	24 ^j^_a_ ± 0.00	23 ^j^_b_ ± 0.00	24 ^h^_a_ ± 0.00	18 ^d^_d_ ± 0.00	19 ^k^_c_ ± 0.00	11 ^i^_f_ ± 0.00	17 ^e^_e_ ± 0.00	24 ^j^_a_ ± 0.00
M4/Van ^fg^	30 ^e^_a_ ± 0.00	25 ^h^_c_ ± 0.05	24 ^h^_d_ ± 0.00	12 ^h^_g_ ± 0.00	24 ^f^_d_ ± 0.00	15 ^e^_e_ ± 0.05	14 ^h^_f_ ± 0.00	27 ^g^_b_ ± 0.10
M5/Gen ^fg^	23 ^k^_b_ ± 0.05	23 ^j^_b_ ± 0.00	26 ^f^_a_ ± 0.00	14 ^f^_c_ ± 0.00	26 ^e^_a_ ± 0.05	9 ^k^_e_ ± 0.00	11 ^j^_d_ ± 0.00	26 ^h^_a_ ± 0.00
M6/Oxy ^efg^	29 ^f^_b_ ± 0.05	24 ^i^_d_ ± 0.00	25 ^g^_c_ ± 0.05	14 ^f^_f_ ± 0.00	23 ^g^_e_ ± 0.05	12 ^h^_g_ ± 0.00	11 ^j^_h_ ± 0.00	32 ^c^_a_ ± 0.00
M7/Oxy ^g^	24 ^j^_c_ ± 0.00	20 ^l^_e_ ± 0.00	23 ^i^_d_ ± 0.00	13 ^g^_g_ ± 0.05	26 ^e^_b_ ± 0.00	14 ^f^_f_ ± 0.00	14 ^h^_f_ ± 0.05	29 ^e^_a_ ± 0.00
M8/Neo ^b^	33 ^b^_a_ ± 0.00	32 ^b^_b_ ± 0.00	33 ^b^_a_ ± 0.10	22 ^b^_d_ ± 0.00	31 ^b^_c_ ± 0.00	21 ^b^_e_ ± 0.05	22 ^b^_d_ ± 0.00	33 ^b^_a_ ± 0.00
M9/Neo ^efg^	26 ^h^_b_ ± 0.05	23 ^j^_c_ ± 0.00	26 ^f^_b_ ± 0.05	11 ^i^_e_ ± 0.00	29 ^c^_a_ ± 0.00	9 ^k^_f_ ± 0.05	19 ^d^_d_ ± 0.00	23 ^k^_c_ ± 0.00
M10/Tetra ^de^	24 ^j^_c_ ± 0.00	22 ^k^_d_ ± 0.05	26 ^f^_b_ ± 0.00	21 ^c^_e_ ± 0.00	31 ^b^_a_ ± 0.00	17 ^c^_f_ ± 0.00	16 ^f^_g_ ± 0.05	26 ^h^_b_ ± 0.05
M11/Kan ^efg^	29 ^f^_b_ ± 0.00	30 ^d^_a_ ± 0.05	28 ^e^_c_ ± 0.00	12 ^h^_g_ ± 0.05	22 ^h^_e_ ± 0.05	9 ^k^_h_ ± 0.00	14 ^h^_f_ ± 0.00	23 ^k^_d_ ± 0.05
M12/Kan ^fg^	25 ^i^_c_ ± 0.00	26 ^g^_b_ ± 0.00	31 ^c^_a_ ± 0.00	14 ^f^_g_ ± 0.05	20 ^j^_e_ ± 0.00	14 ^f^_g_ ± 0.00	17 ^e^_f_ ± 0.00	23 ^k^_d_ ± 0.05
M13/Str ^ef^	25 ^i^_c_ ± 0.05	25 ^h^_c_ ± 0.00	28 ^e^_b_ ± 0.00	16 ^e^_e_ ± 0.00	23 ^g^_d_ ± 0.00	10 ^j^_g_ ± 0.05	14 ^h^_f_ ± 0.05	29 ^e^_a_ ± 0.00
M14/Chl ^ef^	28 ^g^_a_ ± 0.00	28 ^e^_a_ ± 0.00	24 ^h^_c_ ± 0.00	14 ^f^_e_ ± 0.00	26 ^e^_b_ ± 0.05	11 ^i^_f_ ± 0.00	15 ^g^_d_ ± 0.00	26 ^h^_b_ ± 0.00
M15/Amo ^a^	36 ^a^_a_ ± 0.00	33 ^a^_c_ ± 0.00	35 ^a^_b_ ± 0.00	24 ^a^_e_ ± 0.05	33 ^a^_c_ ± 0.00	25 ^a^_d_ ± 0.00	24 ^a^_e_ ± 0.00	36 ^a^_a_ ± 0.00
M16/Ery ^d^	25 ^i^_c_ ± 0.00	31 ^c^_a_ ± 0.05	28 ^e^_b_ ± 0.05	18 ^d^_f_ ± 0.00	21 ^i^_d_ ± 0.00	16 ^d^_g_ ± 0.00	20 ^c^_e_ ± 0.00	28 ^f^_b_ ± 0.05
M17/Ery ^c^	32 ^c^_a_ ± 0.05	27 ^f^_d_ ± 0.00	30 ^d^_c_ ± 0.00	14 ^f^_f_ ± 0.00	31 ^b^_b_ ± 0.00	14 ^f^_f_ ± 0.05	15 ^g^_e_ ± 0.00	31 ^d^_b_ ± 0.00

* Means, followed by different superscripts (within columns) and different subscripts (within rows) are significantly different (*p* < 0.05).

**Table 8:** Antagonistic activity of mutant strain *Br. borstelensis* 10 against test bacteria.

Mutant Strains	Inhibition Zones (mm) of Test Pathogenic Bacteria							
	*B. cereus*_b_	*B. subtilis*_cd_	*E. coli*_a_	*S. typhi*_f_	*K. pneumonia*_e_	*S. aureus*_g_	*M. luteus*_de_	*P. aeruginosa*_bc_
Parent ^f^	23 ^k^_b_ ± 0.00	19 ^k^_e_ ± 0.05	25 ^k^_a_ ± 0.00	18 ^l^_f_ ± 0.05	19 ^h^_e_ ± 0.00	11 ^i^_g_ ± 0.05	20 ^m^_d_ ± 0.00	21 ^h^_c_ ± 0.00
* B1/Van ^e^	27 ^g^_a_ ± 0.10	23 ^h^_d_ ± 0.00	27 ^i^_a_ ± 0.00	23 ^i^_d_ ± 0.00	26 ^e^_b_ ± 0.00	13 ^h^_f_ ± 0.00	22 ^l^_e_ ± 0.00	25 ^f^_c_ ± 0.05
B2/Van ^e^	24 ^j^_c_ ± 0.00	24 ^g^_c_ ± 0.05	29 ^g^_a_ ± 0.00	26 ^f^_b_ ± 0.00	23 ^g^_d_ ± 0.05	16 ^e^_e_ ± 0.05	23 ^k^_d_ ± 0.00	26 ^e^_b_ ± 0.00
B3/Gen ^bcd^	28 ^f^_c_ ± 0.05	29 ^e^_b_ ± 0.00	32 ^e^_a_ ± 0.00	19 ^k^ ± 0.02	24 ^f^_e_ ± 0.05	18 ^c^_f_ ± 0.00	27 ^g^_d_ ± 0.05	28 ^d^_c_ ± 0.00
B4/Gen ^bcd^	32 ^d^_b_ ± 0.00	22 ^i^_g_ ± 0.00	34 ^c^_a_ ± 0.05	28 ^d^_c_ ± 0.00	27 ^d^_d_ ± 0.00	13 ^h^_h_ ± 0.00	23 ^k^_f_ ± 0.00	26 ^e^_e_ ± 0.05
B5/Neo ^bcd^	26 ^h^_e_ ± 0.00	32 ^c^_a_ ± 0.00	31 ^f^_b_ ± 0.00	25 ^g^_f_ ± 0.05	29 ^c^_c_ ± 0.00	16 ^e^_h_ ± 0.05	28 ^f^_d_ ± 0.00	23 ^g^_g_ ± 0.00
B6/Neo ^d^	26 ^h^_c_ ± 0.00	25 ^f^_d_ ± 0.00	28 ^h^_b_ ± 0.00	21 ^j^_e_ ± 0.05	26 ^e^_c_ ± 0.00	13 ^h^_f_ ± 0.00	26 ^h^_c_ ± 0.05	34 ^b^_a_ ± 0.00
B7/Neo ^a^	36 ^a^_a_ ± 0.05	34 ^a^_b_ ± 0.00	36 ^a^_a_ ± 0.00	33 ^a^_c_ ± 0.00	23 ^g^_d_ ± 0.05	20 ^b^_e_ ± 0.05	34 ^a^_b_ ± 0.00	36 ^a^_a_ ± 0.00
B8/Tetra ^d^	25 ^i^_d_ ± 0.00	29 ^e^_c_ ± 0.00	33 ^d^_a_ ± 0.05	20 ^k^_f_ ± 0.00	24 ^f^_e_ ± 0.00	15 ^f^_g_ ± 0.00	24 ^j^_e_ ± 0.00	31 ^c^_b_ ± 0.00
B9/Rif ^bc^	25 ^i^_f_ ± 0.00	31 ^d^_b_ ± 0.05	26 ^j^_e_ ± 0.00	29 ^c^_c_ ± 0.05	32 ^a^_a_ ± 0.00	13 ^h^_h_ ± 0.05	24 ^j^_g_ ± 0.00	28 ^d^_d_ ± 0.05
B10/Rif ^b^	29 ^e^_c_ ± 0.00	34 ^a^_a_ ± 0.05	27 ^i^_d_ ± 0.05	24 ^h^_g_ ± 0.05	31 ^b^_b_ ± 0.00	14 ^g^_h_ ± 0.00	25 ^i^_f_ ± 0.00	26 ^e^_e_ ± 0.05
B11/Poly ^a^	34 ^b^_b_ ± 0.00	33 ^b^_c_ ± 0.00	35 ^b^_a_ ± 0.00	32 ^b^_d_ ± 0.00	32 ^a^_d_ ± 0.05	22 ^a^_e_ ± 0.00	32 ^b^_d_ ± 0.05	34 ^b^_b_ ± 0.00
B12/Poly ^cd^	33 ^c^_a_ ± 0.05	23 ^h^_f_ ± 0.00	32 ^e^_b_ ± 0.05	19 ^k^_g_ ± 0.00	26 ^e^_d_ ± 0.00	17 ^d^_h_ ± 0.05	29 ^e^_c_ ± 0.10	25 ^f^_e_ ± 0.00
B13/Amo ^cd^	27 ^g^_c_ ± 0.05	21 ^j^_g_ ± 0.00	35 ^b^_a_ ± 0.00	25 ^g^_e_ ± 0.00	26 ^e^_d_ ± 0.02	16 ^e^_h_ ± 0.00	30 ^d^_b_ ± 0.00	23 ^g^_f_ ± 0.05
B14/Stre ^bcd^	24 ^j^_f_ ± 0.00	25 ^f^_e_ ± 0.05	35 ^b^_a_ ± 0.00	27 ^e^_d_ ± 0.05	23 ^g^_g_ ± 0.00	15 ^f^_h_ ± 0.00	31 ^c^_b_ ± 0.00	28 ^d^_c_ ± 0.00

* Means, followed by different superscripts (within columns) and different subscripts (within rows) are significantly different (*p* < 0.05).

## Data Availability

Not applicable.
